# Image based modeling of bleb site selection

**DOI:** 10.1038/s41598-017-06875-9

**Published:** 2017-07-27

**Authors:** Sharon Collier, Peggy Paschke, Robert R. Kay, Till Bretschneider

**Affiliations:** 1 MOAC Doctoral Training Centre, University of Warwick, Coventry, CV4 7AL UK; 2 Medical Research Council Laboratory of Molecular Biology, Cambridge, CB2 0QH UK; 3 Department of Computer Science, University of Warwick, Coventry, CV4 7AL UK

## Abstract

Cells often employ fast, pressure-driven blebs to move through tissues or against mechanical resistance, but how bleb sites are selected and directed to the cell front remains an open question. Previously, we found that chemotaxing *Dictyostelium* cells preferentially bleb from concave regions, where membrane tension facilitates membrane-cortex detachment. Now, through a novel modeling approach based on actual cell contours, we use cell geometry to predict where blebs will form in migrating cells. We find that cell geometry alone, and by implication, physical forces in the membrane, is sufficient to predict the location of blebs in rounded cells moving in a highly resistive environment. The model is less successful with more polarized cells moving against less resistance, but can be greatly improved by positing a front-to-back gradient in membrane-cortex adhesion. In accord with this prediction, we find that Talin, which links membrane and cortex, forms such a front-to-back gradient. Thus our model provides a means of dissecting out the role of physical forces in controlling where blebs form, and shows that in certain circumstances they could be the major determining factor.

## Introduction

In migrating cells, protrusions can be driven by the polymerization of actin, such as in lamellipodia and filopodia, or by myosin-II dependent intracellular pressure, resulting in blebs^1, 2^. Blebs arise when the membrane locally detaches from the cortex and bulges outwards due to the flow of pressurized cytosol through the porous cortex^3, 4^. The resulting hemi-spherical membrane protrusion leaves the cortex behind as an F-actin scar, which is degraded whilst a new cortex forms at the blebbed membrane^5, 6^. Activated myosin-II at the new cortex can result in bleb retraction, however, when used as a migratory mechanism, blebs are often not retracted, and thus generate sustained forward motion of the cell body^1, 7^.

*Dictyostelium* cells can use both modes of migration, with blebbing efficiently induced by mechanically resistive environments. Such environments can be imposed by forcing cells to chemotax through microchannels^8^ or by applying uniaxial pressure to them^9^, or as used in this paper, forcing them to move underneath a thin agarose overlay. Under agar assays were initially developed for mammalian cells^10^ and while mimicking some aspects of cell migration in complex 3D environments, such as the increased mechanical resistance, they have the advantage of optical simplicity. In addition, because the cells are flattened under agarose, the analysis of migration is easier allowing us to restrict the analysis of protrusive behavior to two dimensions. Increasing the agarose concentration of the gel from 0.7% to 2% reliably increases the rate of blebbing by *Dictyostelium* cells, yielding a transition from mainly F-actin driven migration, to migration using blebs alone^11^.

If blebbing is to be used for cell movement, there must exist some mechanism to direct bleb formation to the front of cells. That such a mechanism exists is clearly apparent in *Dictyostelium* cells chemotaxing to cyclic-AMP under an agarose overlay, where blebs form preferentially up-gradient. A variety of mechanisms can be envisioned to direct blebbing to the front of the cell: cortical weakening; global pressure gradients; differences in membrane-cortex linker density; and finally cell geometry.

In certain cells, blebs have been reported to occur as a result of weakening and subsequent rupture of the cortex^12^. The clear difference in actin density between the front and rear of the cell has therefore been suggested to lead to increased blebbing at the front, giving a mechanism for bleb site selection^13^. In *Dictyostelium* cells however, blebs form without a loss of cortical integrity, as is shown by the presence of an intact F-actin scar^5^ left behind after the blebs forms. Rupture of the cortex therefore seems an unlikely mechanism to trigger bleb formation in this case.

Contraction of the cortex, producing a pressure surge might also promote blebbing. Myosin-II contractility is largely responsible for pressurizing the cytosol, and indeed, global blebbing propensity does depend on myosin-II activity, with both heavy and light chain myosin-II-null mutants being unable to bleb^11^. Transient pressure gradients are possible thanks to the poroelastic nature of the cytoplasm, which consists of a porous actin meshwork through which viscous cytosol can flow^14, 15^. The uropod is generally the most myosin-II-rich area of *Dictyostelium* cells and is believed to be the most contractile. The high myosin-II contractility in the rear can induce global pressure gradients resulting in forward cytoplasmic streaming. If local pressure surges were to induce local blebbing, this would be predicted to occur in the rear of the cell, which is not observed, except in the case of Formin-A mutants where the cortex of the uropod is weakened^13^.

The plasma membrane is attached to the underlying F-actin cortex by linker proteins, such as those carrying FERM domains, and there is evidence that asymmetric linker distributions can inhibit blebbing in the rear of the cell, and thus maintain cellular polarity^16^. Round blebbing melanoma cells have a uropod like region, which is rich in the linker protein ezrin and where blebbing is significantly reduced, suggesting that ezrin hinders membrane detachment^17^. In chemotaxing *Dictyostelium* cells, the linker protein Talin is enriched in the cell rear and Talin null mutants have an increased blebbing propensity and produce long trailing tails devoid of F-actin, indicating complete detachment of membrane and cortex^18^. Localizations of ezrin and Talin were both shown to depend strongly on myosin-II activity, which is high in the rear of polarised migrating cells^18^. Indeed, in both cases it was shown that myosin-II was required for the posterior localization of the linker proteins. In micropipette aspiration assays, the work required per area to break the linkers is ~63% higher at the rear of wild-type cells compared to the leading edge^19^, but this difference is abolished in Talin null cells. In addition, a recent theoretical study aiming to model the migrational mode changes for cells moving through different matrix environments, found that in order to represent the experimentally observed dynamics, higher membrane to cortex linkage was required at the cell rear^20^.

Finally, membrane geometry can play a key role in determining where blebs form, with blebs preferentially nucleating in regions of negative curvature^21^. Here, membrane tension provides an outwards force, which helps to detach the membrane from the cortex, contrary to convex regions where membrane tension pulls the membrane towards the cortex. We previously showed that the influence of curvature on bleb site selection allows blebs and pseudopods to cooperate, contributing to protrusions clustering at the front of chemotaxing cells. Pseudopods are tightly localized to the leading edge (up-gradient), with a narrow angular distribution, while blebs tend to form on either side of them from the highly concave regions on their flanks. However, regions of negative curvature (high blebbing likelihood) also exist, where blebs do not nucleate, such as on the flanks of the uropod.

In the current paper we investigate the conditions under which cell geometry alone is sufficient to predict where blebs will form, and where this is not the case, what other mechanisms could be responsible.

## Results

### Initialising a biophysical model for blebbing using cell contours extracted from image data

In previous work, Tyson *et al*. described a physical model using artificial cell geometries that showed how pseudopods can induce lateral blebs^21^. The model is described in detail in Materials and Methods. In the current paper, we quantitatively fit this model to complex experimental image data using real cell contours, and so predict where blebs will form.

**Figure 1:**
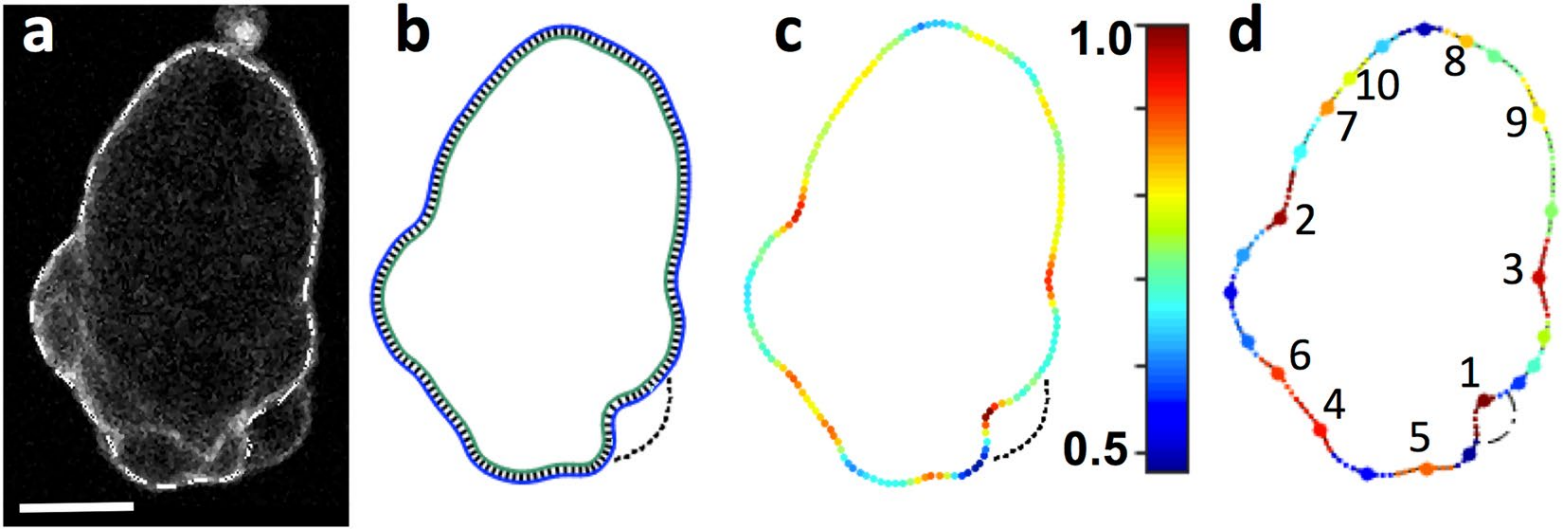
Image based model fitting of blebbing in a *Dictyostelium* cell chemotaxing under 2% agarose. (**a**) Blebs leave behind an F-actin scar labelled by ABD-GFP, indicating the position of the old cortex. Dashed line: Cell contour prior to formation of the bleb to the lower right. Scale bar: 5 μm. (**b**) Initialisation of the model based on the contour in a, with equally spaced linkers positioned between membrane and cortex. Linkers are scaled by a factor of 5 for visual purposes. Dashed line: Position of bleb observed in (**a**). (**c**) Simulation output at sub-critical pressure, which is not sufficient to break linkers and produce blebs. Colours indicate the distribution of linker lengths, from dark blue (relaxed) to dark red (maximally extended), as indicated by the colour bar. Red regions are more likely to nucleate blebs under increased pressure. (**d**) First 10 predicted bleb nucleation sites, ranked in order of likelihood of linkers breaking. Dash-dot line: Outline of most likely model bleb computed at critical pressure.

Cell contour coordinates were extracted from *Dictyostelium* cells chemotaxing to cyclic-AMP under 0.7% and 2% agarose (low and high resistive environments). The cells express an F-actin marker, ABD-GFP, but since the blebbed membrane is initially devoid of actin, fluorescent Rhodamine dextran was added to the agarose gel to act as a negative stain. We use QuimP segmentation software^22^ to determine cell membrane coordinates from the fluorescence images (Fig. 1a).

The basic framework of the model is depicted in Fig. 1b with the cell membrane (blue) coupled to the cell cortex (green) through cortex-membrane linkers (black connecting lines). To initialize the model, cell contours are divided into equidistant intervals, defining nodes on the membrane with an average spacing of 0.3 μm. The actin cortex is defined as a closed contour, consisting of the same number of nodes N, which is a distance *d* away from the membrane, where *d* is the average membrane-cortex linker length (30 nm). Linkers are then positioned between every membrane and cortex node pair. For any given contour, at *t* = *0* in the simulation, linkers are distributed homogeneously, thus eliminating any effects curvature may have on their distribution.

Linkers are described in a coarse-grained way so that each of them must be understood as reflecting the behavior of an ensemble average of linkers with a certain density. This simplification is necessary, because our main focus is on the selection of bleb sites rather than the exact nucleation mechanism itself, and for computational feasibility. We determined the numerical robustness of our model to changes in linker spacing and found that reducing the average spacing to 0.15 *μm* and 0.075 *μm* did not change the order of the dominant bleb nucleation sites (Figure SI 1). A more refined model, which explores nucleation mechanisms in detail, can be found in ref. 23, where the unbinding of individual linkers is not only force-dependent, but also stochastic.

### The strain on cortex-membrane linkers translates into local blebbing propensity

To assess the quality of bleb site predictions, a measure is required for the likelihood of bleb nucleation in a given region, relative to other regions on the same contour. Blebbing propensity was determined by computing the evolution of membrane nodes in the model, and changes in linker lengths, according to the governing equations (eqs 1 and 2). The intracellular pressure has a significant effect on blebbing propensity globally, in line with observations of increased blebbing of cells in hypotonic medium. High values for the model pressure produce many blebs, whilst at low enough pressures, no blebs will nucleate. To determine local blebbing propensity, we perform computations at a sub-critical pressure, which we define as the highest pressure that does not result in linkers breaking and subsequent nucleation of blebs. This reveals regions where linkers are most extended, and therefore more likely to break and nucleate a bleb (Fig. 1c). We determine local maxima in the heat maps of linker lengths and rank these sites in order of their bleb nucleation likelihood: The model’s most likely bleb site corresponds to the longest linker, and is given a rank of *1*, the least likely nucleation site has a rank of *n*, where *n* is the number of sites that exist for a given cell. The local maxima in the linker length distribution computed at the sub-critical pressure yield single node positions for bleb nucleation sites, whilst the width of each bleb site is determined by the distance between the first two local minima on either side of the nucleation site (Fig. 1d). We demonstrate in Figure SI 2(a–f) that the order of model bleb sites predicted by linker length does indeed correspond to the order blebs nucleate in upon increasing the hydrostatic pressure parameter of the system above the critical value. The nucleation of multiple blebs in a single time frame is shown both experimentally and in the model in Figure SI 3(a,b).

The sub-critical pressure varies from time point to time point; in Figure SI 4 we see a clear separation between the observations for cells migrating under 2% and 0.7% agarose. Cells under 0.7% agarose are typically more polarised, having circularities closer to 0, and bleb at lower pressures. Cells migrating under 2% agarose show significantly less variance in both the sub-critical pressure and circularity compared to 0.7% data, indicating that these cells are operating close to the limit of intracellular pressure.

### Cell geometry in the model is a good predictor of bleb sites for 2% agarose, but not for 0.7%

**Figure 2:**
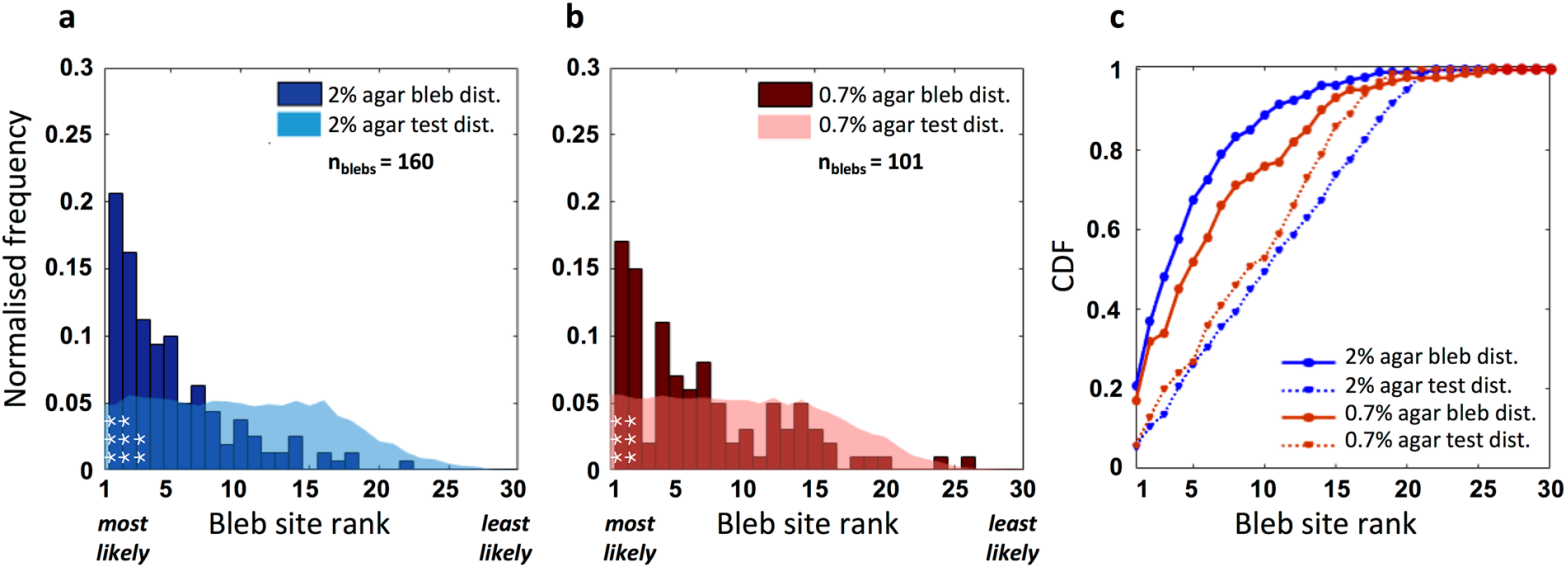
Bleb sites are not distributed randomly. (**a**,**b**) Frequency of observed blebs plotted against model bleb site rank, with test distributions assuming blebs are randomly distributed shown in lighter colours (Blue: 2% agarose, 160 blebs across 8 cells; red: 0.7% agarose, 101 blebs across 13 cells). Both distributions are strongly weighted towards the most likely ranked sites predicted by the model, where differences to the test distributions are significant on a level of 0.001 (bins marked ***, χ^2^ test, class widths of 1 with Bonferroni corrections for multiple comparisons). (**c**) Cumulative distribution function (CDF) demonstrate that the majority of experimentally observed bleb sites are captured within a relatively small proportion of model likelihood ranks, and are clearly not distributed randomly. The CDF curve for 2% agarose is shifted up and to the left compared to the 0.7% agarose data (under-curve areas of 82.48% and 75.60%, respectively), showing that the original model predicts bleb site selection in a highly resistive environment better (Theoretical maximum: 100% if all observed blebs would be predicted by the most likely model bleb site).

To predict the spatial distribution of blebs, we automatically track the positions of experimentally observed blebs, and use the contours from time frames immediately preceding nucleation events as model input. For a given contour, we record the model bleb site ranking at the position of the experimental bleb observed in the next time frame. This procedure was repeated for 160 blebs observed in 133 time frames of 8 migrating cells under 2% agarose and 101 blebs in 100 time frames of 13 cells under 0.7%. Figure 2(a,b) shows the frequency distribution of model ranks for experimentally observed blebs (bleb rank distribution, or short, bleb distribution). A test distribution is generated by assigning nucleation sites randomly, giving the distribution to be expected if all bleb sites were equally likely. Bleb and test distributions for cells migrating under either 2% or 0.7% agarose are shown in Fig. 2a and b. χ^2^ tests were used to compare the distributions, with class widths of 1, and Bonferroni correction for multiple comparisons.

The predicted bleb distributions for both data sets were significantly better than the test distributions (p-values: 3 * 10^−25^ for 2%; 8 * 10^−6^ for 0.7%), with the 2% data having a greater number of significant bins than the 0.7% data. In addition, for both agarose concentrations, the highest frequency of experimental blebs coincides with the highest scoring bleb site in the model. Cumulative distribution function (CDF) plots show that the majority of real bleb sites are captured within the top few most highly ranked bleb sites produced by the model (Fig. 2c). Comparing the areas under the CDF curve for each dataset to the area of the theoretical maximum, where 100% of the experimental blebs observed nucleate from the most likely model site, enables us to estimate the predictive power of our model. For cells migrating under 2% agarose, this area is 82.37% of the theoretical maximum area, compared to 75.60% for the 0.7% agarose data.

Our results therefore show that cell geometry is a good predictor of where blebs will form in chemotaxing *Dictyostelium* cells, especially for cells migrating in highly resistive environments.

### In the model, a rounded cell rear under 2% agarose is sufficient to decrease blebbing propensity at the rear, but elongated cells under 0.7% agarose must possess an additional mechanism

**Figure 3:**
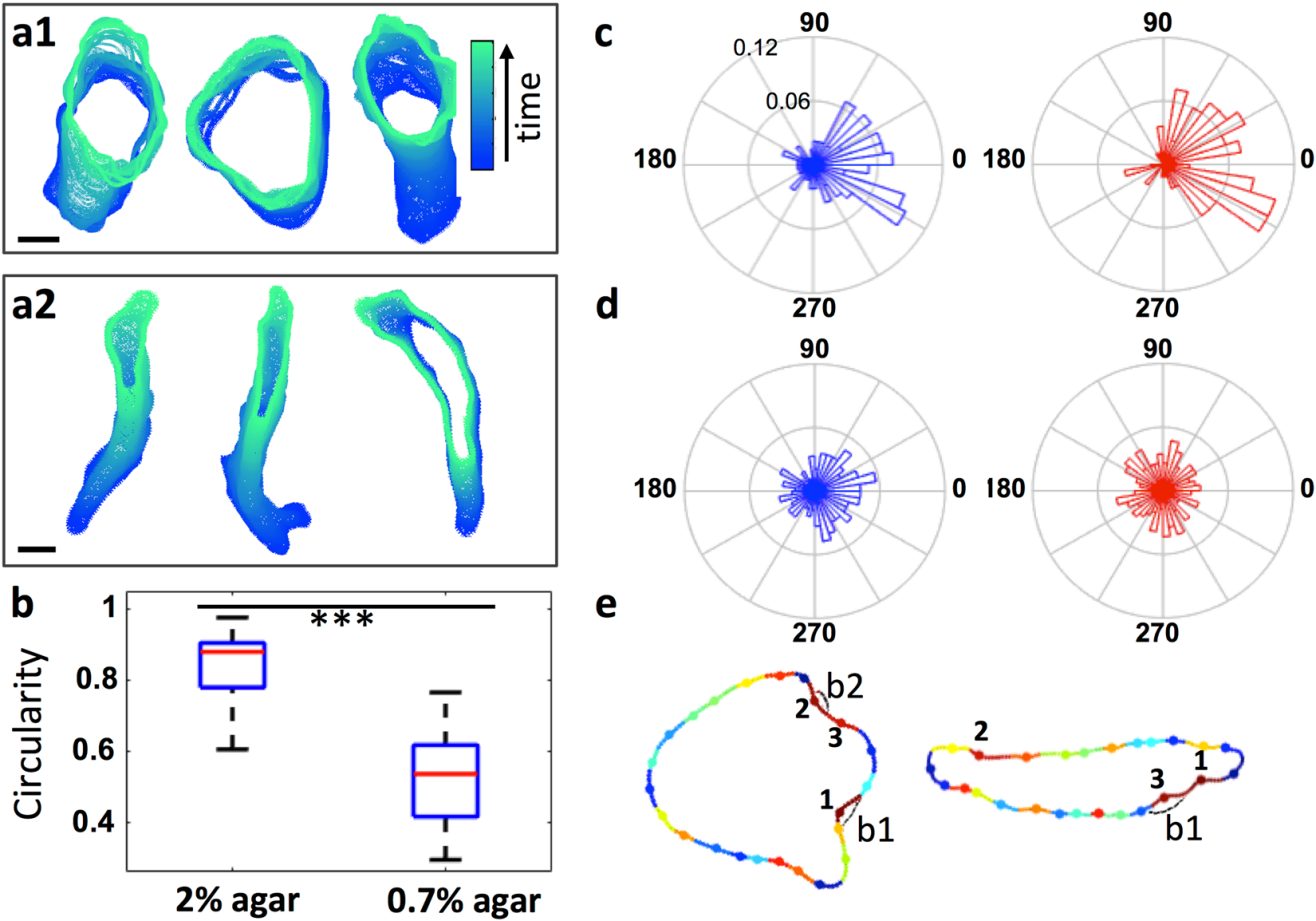
Observed front-rear gradients in blebbing are predicted by the original model for cells under 2% agarose, where blebs are suppressed at the round cell rear. In elongated cells under 0.7% agarose additional mechanisms are required. Contours of cells migrating under 2% agarose cells are rounder (**a1**), under 0.7% agarose more elongated (**a2**), with differences in circularity being significant (**b**) Mann Whitney U-test, p value < 0.001. (**c**) Polar histograms of the angular position of experimentally observed bleb nucleation sites around the cell contour, for both 2% agarose (blue) and 0.7% agarose (red), confirm that blebs are more frequently observed in the front half of the cell (74% of all blebs observed under 2% agarose are seen to be confined to the front half of the cells, and 88% under 0.7% agarose). Note that we define the 0°–180° axis to be the direction of the chemotactic gradient, with 0° corresponding to the front of the cell. (**d**) Polar histograms of the top 3 likely bleb sites predicted by the model for both agarose datasets. The original model based on geometry alone is sufficient to generate a gradient in blebbing activity under 2% agarose (blue), with blebbing suppressed at the round cell rear (we obtain an assymetric distribution of blebbing events spatially, with 63.4% of blebs in the front). Under 0.7% agarose (red), however, the distribution of blebbing events is spatially homogeneous (50.3% of the nucleation sites at the front), suggesting additional mechanisms to direct blebs to the front. (**e**) Representative model outputs for a cell under 2% agarose (left) and 0.7% agarose (right) demonstrating this effect: Under 2% agarose the 3 most likely (red) regions are at the front. Under 0.7% agarose red labelled regions can be also seen at the cell rear, contrary to what we observe experimentally.

We next investigated the cases where the model’s predictions break down, especially why it performs better with cells under 2% compared to 0.7% agarose. The movies show two major differences between cells in the two conditions: (i) Cells under 2% agarose are much rounder than those under 0.7% agarose, and in particular have a pronounced round tail (circularity difference p < 0.001, Mann Whitney U test; Fig. 3a1,a2,b); (ii) the chemotactic orientation of experimental blebs is superior in cells migrating under 0.7% agarose, with 88% of blebs nucleating in the front half of the cell compared to 74% under 2% agarose (Fig. 3c).

For 2% agarose, the model predicts 63.4% of blebs to lie in the front half (Fig. 3d). The difference in the number of blebs predicted between the cell front and rear is due to the highly rounded cell rear, whose positive curvature disfavours blebbing.

For the 0.7% agarose data the angular distribution of model blebs is flat with a small dip at the front and the rear of the cell, reflecting high positive curvature in these regions. Because cells are more elongated, the chances of finding regions of negative curvature (likely bleb sites) are more evenly distributed around the cell contour, and thus the model predicts that 50% of blebs will form in the front half. The model is therefore not able to reproduce the strong asymmetry observed in experiments (88% in front). We conclude that there must be an additional mechanism in elongated cells that either restricts blebbing to the cell front, or reduces the effects of negative curvature along the flanks of a cell.

### Talin enrichment is inversely proportional to blebbing

**Figure 4:**
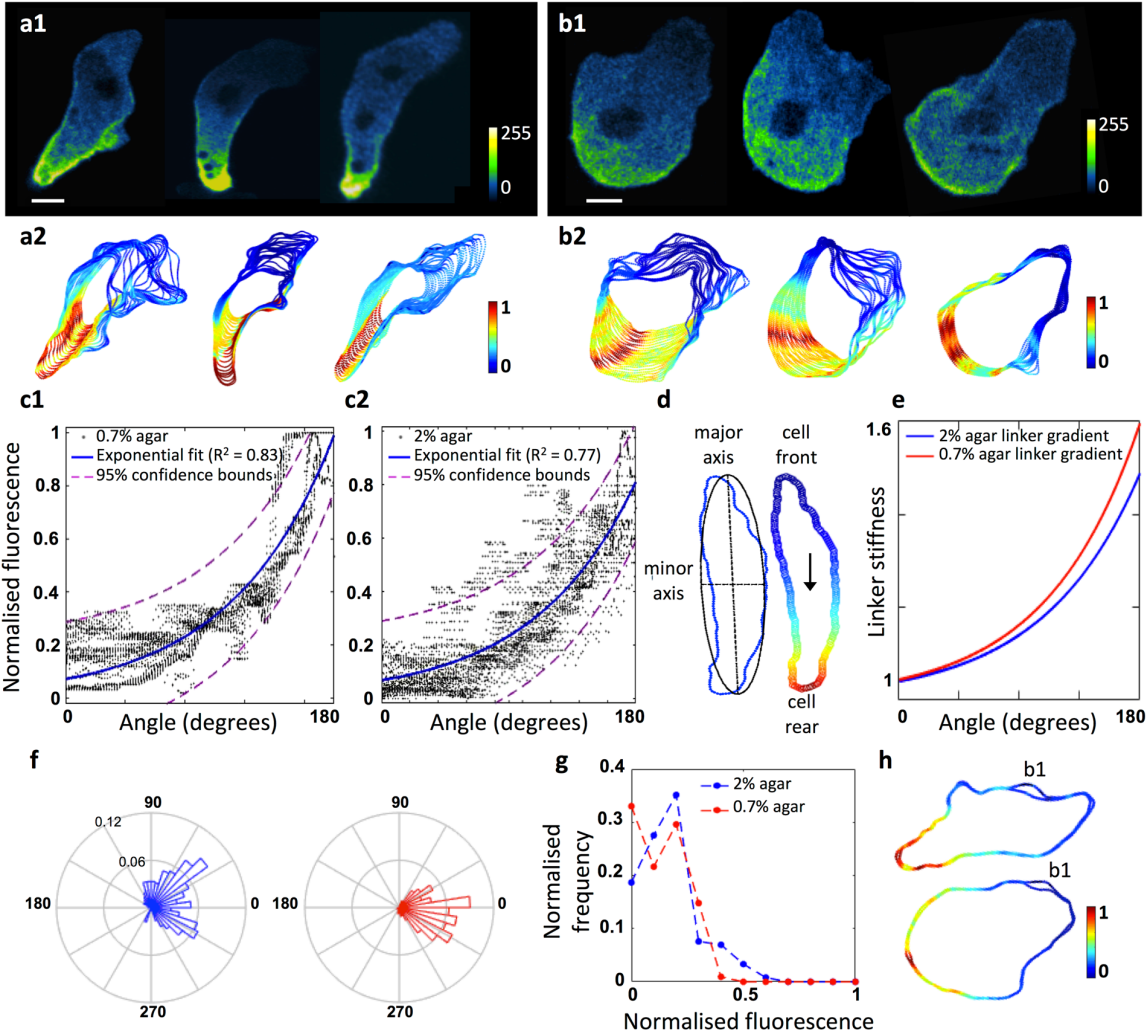
Talin A gradients correlate with front-rear gradients in blebbing activity. (**a1**) *Dictyostelium* cells chemotaxing under 0.7% agarose, with TalA-mNeon primarily enriched in the cell rear. (**a2**) Corresponding cell contours for 30 time points recorded at 2 frames per second, colour scaled according to fluorescent intensity normalised for each cell (blue: low, red: high). (**b1**,**b2**) Graded distribution of TalA-mNeon observed under 2% agarose. Bars: 5 microns. (**c1**,**c2**) Combined fluorescence profiles for the 3 time series shown in (**a2**,**b2**) plotted against angular position around the cell contour, with 0° defined as the cell front. TalA-mNeon fluorescence increases exponentially from cell front to rear (0.7% agarose, R^2^ = 0.83, 2% agarose, R^2^ = 0.77). (**d**–**e**) In the model we translate observed TalA-mNeon distributions into gradients in membrane to cortex linkage strength, along the polarization axis of the cells. We use a maximum of 60% increase in linker strength between the very front and rear of the cells, as measured in micropipette aspiration assays^19^. (**f**) Polar histograms of the angular distribution of experimental blebs, for the cell contours shown in figures (**a2** and **b2**) demonstrate that blebs are indeed directed towards the cell front. (2% agarose data, blue; 0.7% agarose data, red.) (**g**) Histograms for the observed bleb frequency against normalised TalA-mNeon fluorescence clearly show that blebs do not nucleate in the regions of highest TalA-mNeon. (2% agarose rank correlation coefficient, −0.80, 0.7% agarose rank correlation coefficient, −0.85). (**h**) Example contours with automatically detected bleb sites indicated by (**‘b1’**) colour scaled according to fluorescent intensity normalised for each cell (top, 0.7% agarose cell, bottom, 2% agarose cell).

A strong candidate for this additional mechanism directing blebbing to the front of elongated cells is a front to rear gradient of a cortex-to-membrane linker, such as Talin (see Introduction). In line with previous work, we observe that TalA-mNeon is enriched in the posterior of cells chemotaxing under agarose (Fig. 4a1,b1). To analyse this in detail, we segmented cell outlines, and extracted cortical fluorescence intensities, thus allowing us to visualize cell contour plots over time, colour coded according to normalized fluorescence (Fig. 4a2,b2).

Figure 4c1 and c2 show the collective TalA-mNeon fluorescence profiles plotted against the angle along the cell polarization axis (3 cells for each agarose concentration, 30 time points per cell). A striking spatial gradient of TalA-mNeon is observed in both low and high resistive environments, which exponentially increases from front to rear of the cell (R^2^ values for an exponential fit to the data are 0.83 and 0.77 for 0.7% and 2% agarose respectively). Angular histograms for the experimentally observed blebs for the cells shown in Fig. 4a1 and b1 clearly demonstrate that blebs are directed to the cell front where the TalA-mNeon fluorescence is lowest (Fig. 4f). To confirm this further, histograms for the experimental bleb frequency, binned according to TalA-mNeon fluorescence, explicitly show that blebs do not occur in regions of maximal Talin accumulation (Fig. 4g), which we have already seen coincide with the rear of the cells. The blebbing frequency negatively correlates with TalA-mNeon fluorescence, with rank correlation coefficients of −0.80 and −0.85 for the 2% and 0.7% agarose datasets respectively. Examples of the experimental blebs observed are shown in Figure SI 5, whilst examples from the extracted contours with automated bleb detection are shown in Fig. 4h.

Thus we conclude that the areas of highest blebbing activity coincide with areas of lowest Talin and vice-versa. Accordingly, Talin knockouts (*talA/B* null) produce many more blebs than wildtype cells, with these blebs not limited to the cell front (Figure SI 6).

### Inclusion of linker gradients improves model fits for elongated cells

**Figure 5:**
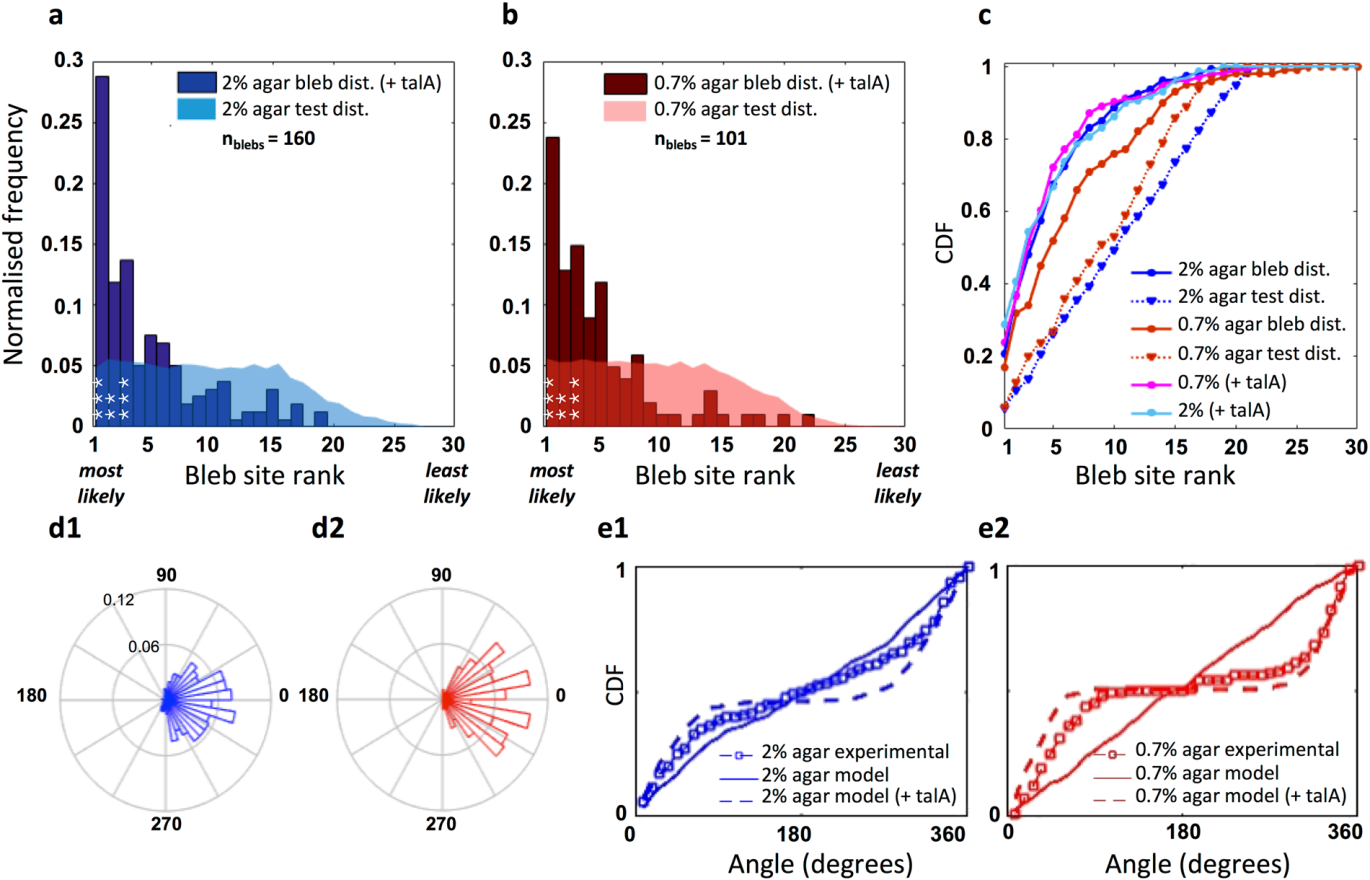
Inclusion of an exponentially graded distribution in cortex membrane linker strength greatly improves model performance for elongated cells chemotaxing in low resistive environments (0.7% agarose). (**a**,**b**) Frequency of observed blebs plotted against model bleb site rank, with corresponding test distributions in lighter colours (Blue: 2% agarose, 160 blebs across 8 cells; red: 0.7% agarose, 101 blebs across 13 cells). Bins marked ***indicate where differences to the test distributions, which assumes blebs are randomly distributed, are significant on a level of 0.001 (χ^2^ test, class widths of 1 with Bonferroni corrections for multiple comparisons). (**c**) Cumulative distribution functions (CDF) demonstrate that including gradients in linker strength result in much improved performance for 0.7% agarose data, with the updated CDF curve shifted up and to the left. A larger proportion of the experimental data is captured within a smaller number of model bleb sites, thus increasing the predictive power. Areas under the curve increase from 75.60% to 83.07% for the 0.7% agarose cells, whereas for 2% cells, the difference is marginal, with under curve areas of 82.37% compared to 82.48%. (**d1**,**d2**) Angular distributions of the 3 most likely bleb sites predicted by the model after inclusion of the gradient in linker stiffness, blebs are now seen to be efficiently directed towards the cell front. (91% and 95% of bleb sites in the cell front for 2% and 0.7% data respectively). (**e1**,**e2**) Cumulative frequency plots of the angular bleb distribution of experimental blebs compared to the top 3 model bleb sites both before and after inclusion of the linker stiffness gradient, for each agarose concentration.

In line with the observed distributions of Talin we modified our model to include an exponential gradient in linker stiffness, increasing from the leading edge to the cell rear (Fig. 4d,e). Figure 5a–c show the resulting bleb distributions and CDF plots for the 2% and 0.7% experimental datasets. For both datasets, the proportion of experimental blebs observed at the models’ most likely bleb site clearly increases. The CDF plots show that the inclusion of the linker gradient greatly improves the 0.7% data, with the area under the CDF curve increasing from 75.60% to 83.07% compared to the theoretical maximum. On the contrary, the already good performance of the geometry-based model on the 2% data remains unchanged.

The spatial distributions of the 3 most likely bleb sites (Fig. 5d1,d2) show that inclusion of the linker gradient efficiently directs the most likely sites of bleb nucleation to the cell front. We next compare the corresponding cumulative angular frequency plots (Fig. 5e1,e2) to the experimental bleb frequency plots, and the model sites predicted by geometry alone. This shows that the initial geometry based model matches the 2% agarose data much better than the 0.7% data (Kuiper test p values for the null hypothesis that the distributions are the same for 2% data compared to the model: 0.005 ≤ p < 0.01, and for 0.7% data compared to the model: 0.001 ≤ p < 0.002. Circular statistics toolbox, MATLAB^24^). Upon inclusion of the linker gradient, the shape of the cumulative frequency of the top 3 model bleb sites for 0.7% agarose much more closely represents the spatial distribution of the experimental blebs (Kuiper test p value for the distributions being the same: p > 0.1).

## Discussion

Migration driven by bleb expansion is characteristic of cell motility in a mechanically resistive or confined environment, where either the small pores or channels through which a cell has to navigate, or high rigidity of the surrounding material, make slow actin-driven protrusions less feasible^25, 26^. The question of how bleb sites are selected, and also restricted around the cell posterior, to aid efficient chemotactic movement in real 3D environments, as opposed to movement on a 2D surface in standard cell cultures, is therefore of great relevance. Previously, simulations of a physical model for artificial cell geometries were presented by Tyson *et al*. to demonstrate how pseudopods can induce lateral blebs^21^. In the current paper, the big step forward is to quantitatively fit this model to complex experimental image data using real cell contours, and also fluorescence distributions of Talin. Cell contour analysis is well established in investigations of cell membrane mechanics, for example in micropipette aspiration^19^ or analysis of cell shape flickering^27^. Our modelling framework is fundamentally different in such that we are able to predict actual cell shape changes, even in migrating cells with highly complex geometries. Combining contour analysis with cortical fluorescence distributions enables us to probe to what extent physical or biological mechanisms could determine the positions of bleb nucleation sites.

In our model, the strain on cortex-membrane linkers is highly dependent on geometry. Whilst other models have also taken into account the influence this strain has on linker dynamics, this has only been considered across small scale local fluctuations of the membrane^23, 28^, whereas our coarse grained approach allows us to study bleb site selection globally, including its dependence on active shape deformations as a result of pseudopod formation. We demonstrate that our approach is applicable to other cell types, by computing how the model fits to data from a *Fundulus* deep cell, and show that the same geometry dependent principles apply, Figure SI 7(a–f).

We show that for cells migrating through mechanically resistive environments, our model based on physical mechanisms alone well describes the location of experimentally observed bleb nucleation sites. However, for cells migrating through less resistive environments, the inclusion of a biological polarization mechanism is required in order to more accurately predict the spatial distribution of bleb sites. We proposed that a likely candidate for preventing blebbing at the cell rear, and thus maintaining cellular polarity is a gradient in cortex-membrane linkage strength, in particular for the linker protein Talin. Although molecular mechanisms responsible for generating graded linker distributions are still unknown, regulation through PIP2, the membrane lipid that binds linkers, has been a recurring theme in current literature^29–31^. In addition, continuous turnover of linkers introduces a stochastic element, possibly accounting for the rare events where blebs have been seen to nucleate in regions of positive curvature.

When cells use blebs for chemotactic movement, the guiding chemotactic gradient must be able to influence where blebs form, directing them preferentially up-gradient. Our results show that this guidance could be exerted quite indirectly through cell geometry. Geometry itself is the product of previous shape changes caused by earlier cellular extensions and retractions, including blebs, F-actin driven pseudopods and spikes, and myosin-II driven retractions. Any or all of these processes could help determine where blebs form, in addition to any direct effect that chemotactic signaling may have on blebbing, such as by locally affecting the membrane-cortex linkers.

New experimental data is required to determine which of these processes is used for steering cells in chemotactic gradients, particularly information about how cells reorientate when the gradient direction is changed. This is however difficult to achieve in under-agarose assays and demands new experimentation techniques such as release of caged chemoattractant through photolysis^32^. The modeling approach described here will be an essential tool in analysing such experiments, but must be extended to account for cortex dynamics. In addition, our approach could be extended to cells moving in 3D through tissues, thanks to recent advances in low phototoxic, fast 3D imaging. However, this will require methods for segmenting and tracking cell shapes accurately in 3D, and the extension of our computational models to 3D.

## Materials and Methods

### A biophysical model for blebbing

We consider a 2D cross section of a cell, with the plasma membrane and cell cortex both approximated by closed contours consisting of a number of discrete nodes, which are connected by linear edges. The spacing between nodes is approximately *0*.*3μm* so that nucleating blebs can be resolved in enough detail.

Corresponding nodes on the membrane and cortex are coupled by cortex membrane linkers, acting as linear springs. Initially, the density and strength of linkers are assumed to be homogeneous around the contour.

Intracellular pressure, acting on the membrane but not the porous cortex, is the main driver of blebbing. It puts linkers under strain, causing them to break beyond a critical extension, and allowing the detached membrane to form a bleb.

In *Dictyostelium* cells, bleb nucleation and expansion are very fast (<0.1 s) compared to cortex remodeling^21^, which is why we treat the cortex as fixed on the timescale of blebbing. A similar approach is used in refs 33 and 34.

Assuming the Helfrich model for membrane bending^35^, the total membrane energy integrated over the normalised contour length is given by,

1 $${E}_{membrane}=\underset{0}{\overset{1}{\oint }}({E}_{tension}+{E}_{bending}+{E}_{coupling}+{E}_{pressure})ds$$

2 $${E}_{membrane}=\underset{0}{\overset{1}{\oint }}(\frac{1}{2}\alpha {(|\frac{dx}{ds}|-{x}_{0})}^{2}+\frac{1}{2}\beta {(|\frac{{d}^{2}x}{d{s}^{2}}|)}^{2}+\frac{1}{2}k{(L-{L}_{0})}^{2}\,+\Delta p)ds$$

where *α*, *β* and *k* define the strength of membrane tension, bending rigidity and linker stiffness respectively. Δp is the difference in pressure across the cell membrane. The resting length of the membrane is denoted by *x*_*0*_ and similarly, *L*_*0*_ is the relaxed linker length. Membrane movements are computed by numerically minimizing the membrane energy using a gradient descent method, with derivatives approximated by finite differences.

The time dependence in the model comes from the inclusion of a viscous drag experienced by a bleb as it expands. For a spherical bleb of radius *R*, expanding with velocity *v*, the Stoke’s drag force is given by,

3 $${F}_{d}=6\pi \eta vR$$

where η is the dynamic viscosity of water.

The model is parameterized as before^21^ (See SI Table 1 for parameter values).

### Cells and microscopy

Ax2 strain of *Dictyostelium discoideum* (R.R.K laboratory strain; DBS0235521) grown on HL5 medium at 22 °C. The cells were transformed with GFP-ABD120, an F-actin marker^36^. The cells were starved for 5.5 h after washing, and pulsed with cyclic AMP every 6 minutes after the first hour, to give a total cAMP concentration of ~90 nM. The under agarose assay was used to induce blebbing. Agarose gel of either 0.7% or 2% agarose in KK_2_ was set onto Lab-Tek coverslips (Thermo Fisher Scientific). Two wells were cut into the agarose, into one, 4 μM cAMP was inserted, to set up a linear gradient across the gel, whilst the cells were inserted into the other well. Rhodamine-B-isothiocyanate-dextran (RITC-Dx) was added to the gel to act as a negative stain, to aid in image segmentation. Images were acquired in both relevant fluorescent channels, at 2 fps, using either a spinning disc confocal microscope (Ultraview; PerkinElmer) with a 100× oil-immersion objective, or with a Zeiss 710 laser scanning confocal microscope with a 63× oil-immersion objective^11^. For Talin experiments: TalA/B- double knockout of FERM domain linker proteins Talin A and Talin B (A kind gift of M.Tsuijioka to R.R.K laboratory, R.R.K laboratory strain HM1554) were transformed with mNeon fused to the C-terminal of the membrane to cortex linker protein Talin A. The Talin A gene was amplified using the primers: oPI_134 (CCGGATCCAAAATGTCAATTTCATTAAAAATTAATATTG) and oPI_135 (GCACTAGTATTTTTATTATAATTTTGTTTTCTTGAATTTAAC) with genomic DNA as template.

oPI_134 generates a BamHI site and oPI_135 a SpeI site. The PCR product was cut with both enzymes and cloned in the BglII/SpeI sites of pPI62 resulting in an in frame fusion with mNeon.

pPI62 is an extrachromosomal plasmid carrying a G418 resistance cassette. The expression of the fusion protein is under the control of an actin15 promotor. The selection of clones was done with 20 µg/ml G418.

### Bleb tracking

For each contour used as a model input, we extract the coordinates of the cell contour at the next time frame, and search for morphological changes. To capture protrusions, we check whether points from the latter time frame lie inside, on the boundary, or outside the contour of the earlier time frame, and only keep those found outside. Since bleb expansion is very fast compared to that of actin driven pseudopodia^11^, we can distinguish which protrusion points are due to blebbing based on a distance constraint; the largest protrusions that occur over a single time frame (for images collected at 2 fps) are only feasible through fast expansion driven by hydrostatic pressure. Regions marked as blebs are then manually checked. All analysis was performed in MATLAB.

### Determining cellular polarization axis

All contours to be analyzed were fitted to an ellipse, using a least squares approach to estimate parameters *a* and *b*, the minor and major axis respectively. The polarization axis of the cell was defined to be the major axis of the ellipse. The leading edge was then defined to be the end of the major axis that corresponded to the direction of persistent movement when compared to five subsequent time frames. The node closest to the major axis at the leading edge side of the cell was defined to be at 0° for circular analysis and statistical testing (rotationally invariant Kuiper test used to compare distributions over a circular domain). The exponential gradient in linker stiffness was applied along the major axis of the cell, from leading edge to the rear (Fig. 3f).

### Data Availability

Original image time series and segmented contour data used in this research are openly available from the University of Warwick Research Archive Portal (WRAP) at http://wrap.warwick.ac.uk/90238.

## Electronic supplementary material

Supplementary Information

Movie 1

Movie 2
