# Supplementary material for: Image based modeling of bleb site selection
Source: Sci Rep. 2017 Jul 27;7:6692. doi: 10.1038/s41598-017-06875-9 (PMC5532237; doi:10.1038/s41598-017-06875-9)
Supplement: Supplementary file 1 — Supplementary Information [file 41598_2017_6875_MOESM1_ESM.pdf]

Supplementary Information to

Image based modelling of bleb site selection

Sharon Collier[1], Peggy Paschke[2], Robert R. Kay[2], Till Bretschneider[3*]

1: MOAC Doctoral Training Centre, University of Warwick, Coventry CV4 7AL, UK
2: Medical Research Council Laboratory of Molecular Biology, Cambridge CB2 0QH, UK
3: Department of Computer Science, University of Warwick, Coventry CV4 7AL, UK

*Corresponding author: T.Bretschneider@warwick.ac.uk

Supplementary Table 1

Parameter	Value	Reference
*Node spacing	0.3 μm	
Fractional contour length, Δs	1/N where N is the number of nodes	
*Membrane stiffness coefficient, α	8.5 * Δs^2 pN/μm	
Membrane bending coefficient, β	0.07 * Δs^4 pN/μm	1
Membrane resting length, x_0	95% of distance between nodes at equilibrium	2
*Linker stiffness coefficient, k	10 pN/μm	
Linker resting length, L_0	40 nm	3
*Linker breaking length	56 nm	
*Linker minimum length	30 nm	

Table SI 1 Model parameters

* Free parameter

References to Table SI 1

1. Lieber, A. D., Yehudai-Resheff, S., Barnhart, E. L., Theriot, J. A. & Keren, K. Membrane

tension in rapidly moving cells is determined by cytoskeletal forces. *Curr. Biol.* **23,** 1409–1417 (2013).
2. Morris, C. E. & Homann, U. Cell surface area regulation and membrane tension. *J. Membr. Biol.* **179,** 79–102 (2001).
3. Kanchanawong, P. *et al.* Nanoscale architecture of integrin-based cell adhesions. *Nature* **468,** 580–4 (2010).

Supplementary Information Figure Legends:

Figure SI 1: (a) Example of the distribution of normalised linker lengths computed at the sub-critical pressure for varying node densities for a single cell contour, colour coded from least extended (blue) to maximally extended (red). (b) First 10 bleb nucleation sites predicted by the model for the same cell contour shown for each corresponding spacing. Order of first three bleb sites remains unchanged. (c) Plot of absolute mean rank difference between coarsest and finest grid spacing, for all contours and all bleb sites, demonstrating minimal re-orderings of ranked bleb sites occur, even upon increasing the model node density four-fold. The least re-orderings are observed for the most likely and least likely model sites.

$$Absolute\ Mean\ Rank\ Difference_i = \frac{\left|\sum_{j=1}^{N}\left(CoarseRank_{i,j} - FineRank_{i,j}\right)\right|}{N}$$

, where *N* is the number of contours, and *i* is the *ith* bleb site for each contour, *j.*

Figure SI 2: Simulated bleb nucleation (a) Model prediction of the likelihood order of regions around the cell contour nucleating a bleb. Bleb sites are ranked based on linker length, with the top 5 sites labelled. Colours indicate the distribution of linker lengths, from dark blue (relaxed) to dark red (maximally extended). (b-c) Simulated bleb nucleation and expansion upon increasing hydrostatic pressure parameter, demonstrating the order of bleb nucleation matches what is predicted based on linker length. The pressure parameters for the simulations are 78Pa, 79Pa, 81Pa, 81.9Pa and 82.5Pa respectively.

Figure SI 3: Model bleb nucleation (a) *Dictyostelium* cell contours are extracted from confocal image data using QuimP segmentation. The cells express ABD-GFP, an F-actin marker. Blebbing regions are seen to leave behind an F-actin scar, where the old cortex remains intact. The cell shown here is migrating under 0.7% agar. The time series indicates the rapid speed of bleb expansion in *Dictyostelium* cells. The nucleation of two blebs is observed experimentally. Scale bars are of length 5 microns. (b) The extracted cell contour is used as the initial geometry in the model, with linkers positioned between the membrane and cortex at equally spaced intervals. All linkers are scaled by a factor of 5 for visual purposes. Model prediction for the sites of bleb nucleation for the given contour, showing two true positive nucleation sites.

Figure SI 4: Relation between hydrostatic pressure and cellular circularity. All simulations are run at the sub-critical pressure, which we define to be the highest pressure possible in the model, whilst still maintaining no bleb nucleation. The sub-critical pressure for 0.7% and 2% agar cell contours is plotted against the cell's circularity. (2% agar: 126 blebs across 8 cells. 0.7% agar: 122 blebs across 13 cells). Cells migrating under 0.7% agar are typically more polarised, since they migrate with mainly F-actin driven pseudopods, and have few blebbing events. Cell contours used in the model that were extracted from cells under 0.7% agar are shown to require a lower pressure to initiate blebbing. For cells migrating under 2% agar, blebs alone are used to extend the cell front, and blebbing events are frequent. These cells are therefore much more rounded, with circularities close to 1. The small variance is both circularity and sub-critical pressure observed for the contours corresponding to 2% agar conditions suggest that these cells are functioning in a close-to-

limit regime. In our previous work, a hydrostatic pressure parameter of 81 Pa was used in the simulation of an artificial, very rounded cell contour. Here, we find the optimum hydrostatic pressure (where model specificity and sensitivity are maximal) for all the contours considered to be in the range 30-100 Pa.

Figure SI 5: Blebs are observed to nucleate in regions of low talin-A (a1-a2) *Dictyostelium* cells chemotaxing under 0.7% agar, expressing a talA-mNeon construct. Blebs are observed to nucleate at the front of the cell, where there is little talin, and not observed to form at the cell rear where talin-A is enriched. (b1-b2) Examples of bleb nucleation in cells chemotaxing under 2% agar. Again, blebs are seen to nucleate in regions where talin-A is not enriched. Images recorded at 2 frames per second. See Supplementary Movies Movie SI 1 and Movie SI 2.

Figure SI 6: Morphology and migration efficiency of talA/B- knockout cells differs greatly from that of wild type Ax2 cells. (a1-a3) Vegetative talA/B- cells in buffer are seen to have a very rounded morphology and do not attach to the substrate. (b1-b3) Early development stage talA/B- cells in buffer still exhibit poor attachment to the substrate. Long bleb-like protrusions are often observed to extend towards neighbouring cells. (c1-c3) Early development stage talA/B- cells under agar. The cells are much flatter due to compression by the agar. Long bleb-like protrusions as well as classic spherical blebs are observed to nucleate very frequently. The migration efficiency of the knockout cells under agar is very poor due to (i) lack of proper rear retraction, (ii) many non-productive blebs at the sides and rear of the cell and (iii) retraction of protrusions leading to multiple blebbing events on the flanks of the retracting region.

Figure SI 7- Image based model fitting approach applied to *Fundulus deep cell*:
Here we fit the bleb site selection model to an image sequence of a migrating *Fundulus* deep cell (courtesy of Rachel Fink, Mount Holyoke College, Holyoke, MA). (a) Bleb and test distributions for the *Fundulu*s deep cell illustrate the same trend in the likelihood of bleb sites as seen for chemotaxing *Dictyostelium* cells, with the distribution for experimentally observed bleb sites being heavily skewed to the most likely regions predicted by the model. (b) Snapshot of the migrating cell showing bleb nucleation in the subsequent time frame, as indicated with an arrow. (c) Ranked bleb region map produced from the model indicates that the experimental bleb site corresponds to the most likely site predicted by our model. (d) CDF curves for the bleb and test distributions for the *Fundulus* deep cells, plotted against *Dictyostelium* data. The bleb site selection model based on geometry alone yields a comparable performance with that of *Dictyostelium* cells migrating in highly resistive environments. (e-f) Boxplot for the circularities of each contour analyzed, and the cell path over time [scale bar 5μm] show a strong resemblance to the *Dictyostelium* cells migrating under 2% agar, both in cell shape and efficiency of directing blebs to the cell front.

Supplementary Information Movie Legends:

Movie SI 1: Talin-A gradients correlate with front-rear gradients in blebbing activity (blue: low, green: high). *Dictyostelium* cells chemotaxing under 0.7% agarose, with talA-mNeon primarily enriched in the cell rear. Corresponding movie to figure 4a1 which includes a colour bar (middle cell). talA/B- cell expressing talA-mNeon. Images recorded at 2 frames

per second.

Movie SI 2: Talin-A gradients correlate with front-rear gradients in blebbing activity (blue: low, green: high). *Dictyostelium* cells chemotaxing under 2% agarose. Corresponding movie to figure 4b1 which includes a colour bar (leftmost cell). TalA/B- cell expressing talA-mNeon. Images recorded at 2 frames per second.

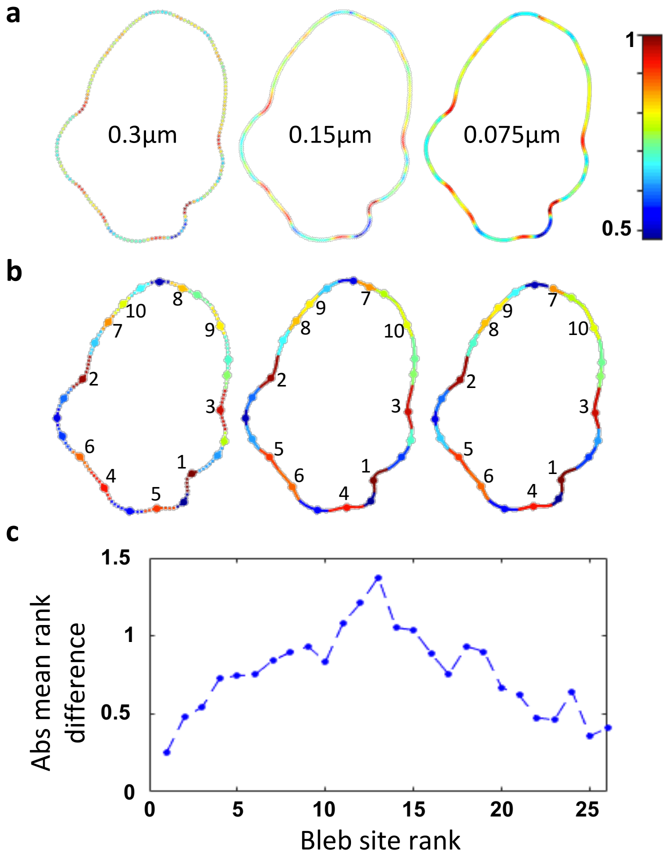

a
0.3µm
0.15µm
0.075µm
1
0.5
b
c
Abs mean rank difference
Bleb site rank

Figure SI 1

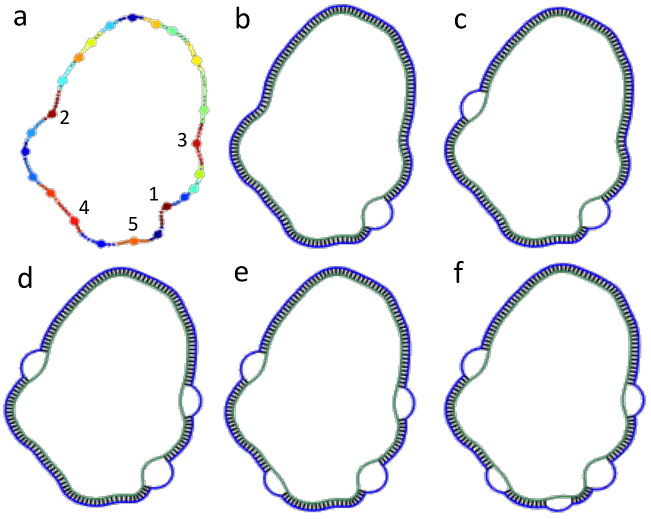

Figure SI 2

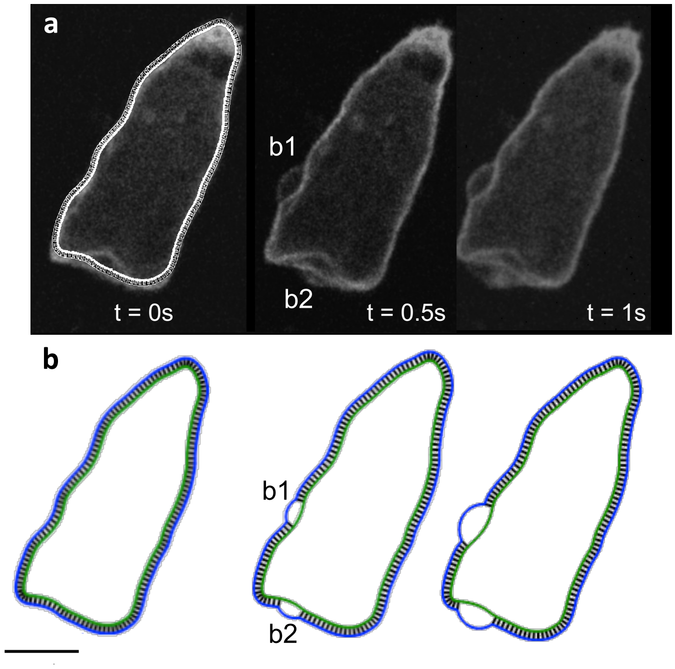

Figure SI 3

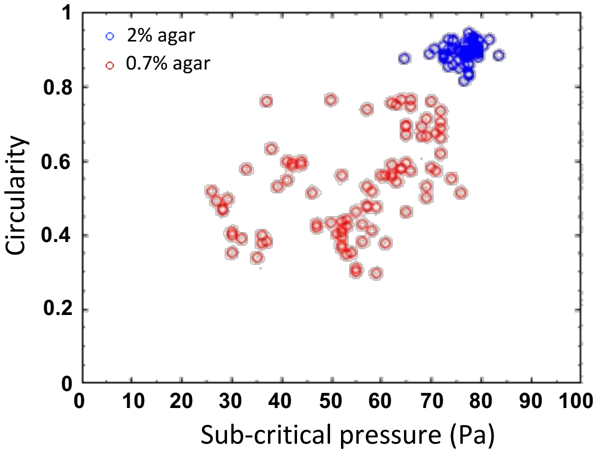
2% agar
0.7% agar
Circularity
Sub-critical pressure (Pa)
0
0.2
0.4
0.6
0.8
1
0
10
20
30
40
50
60
70
80
90
100

Figure SI 4

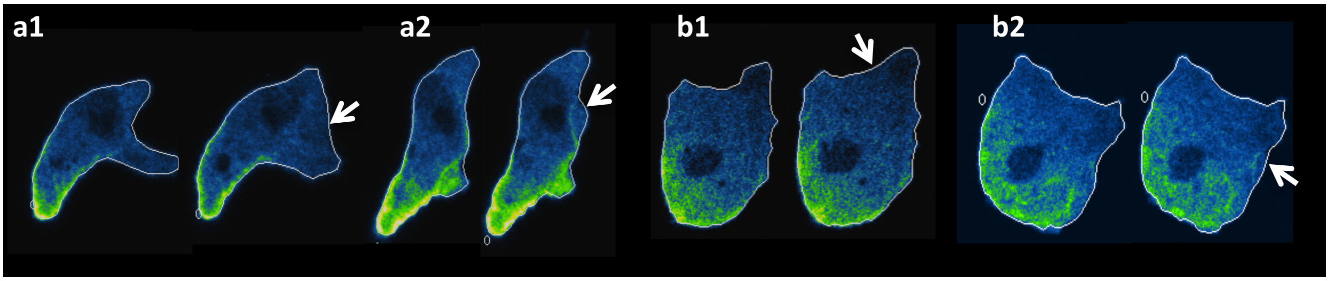

Figure SI 5

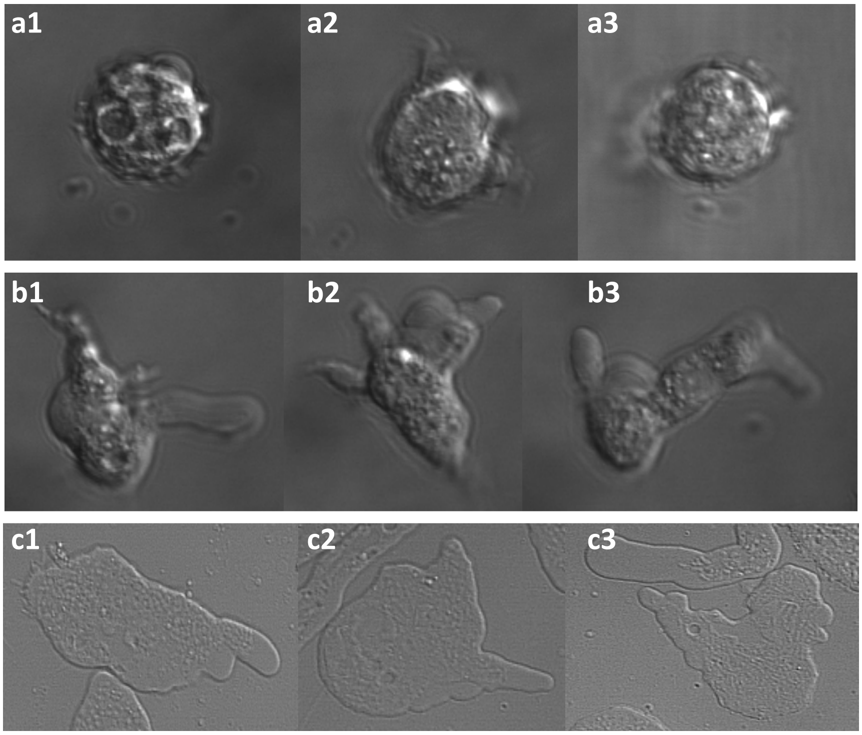

Figure SI 6

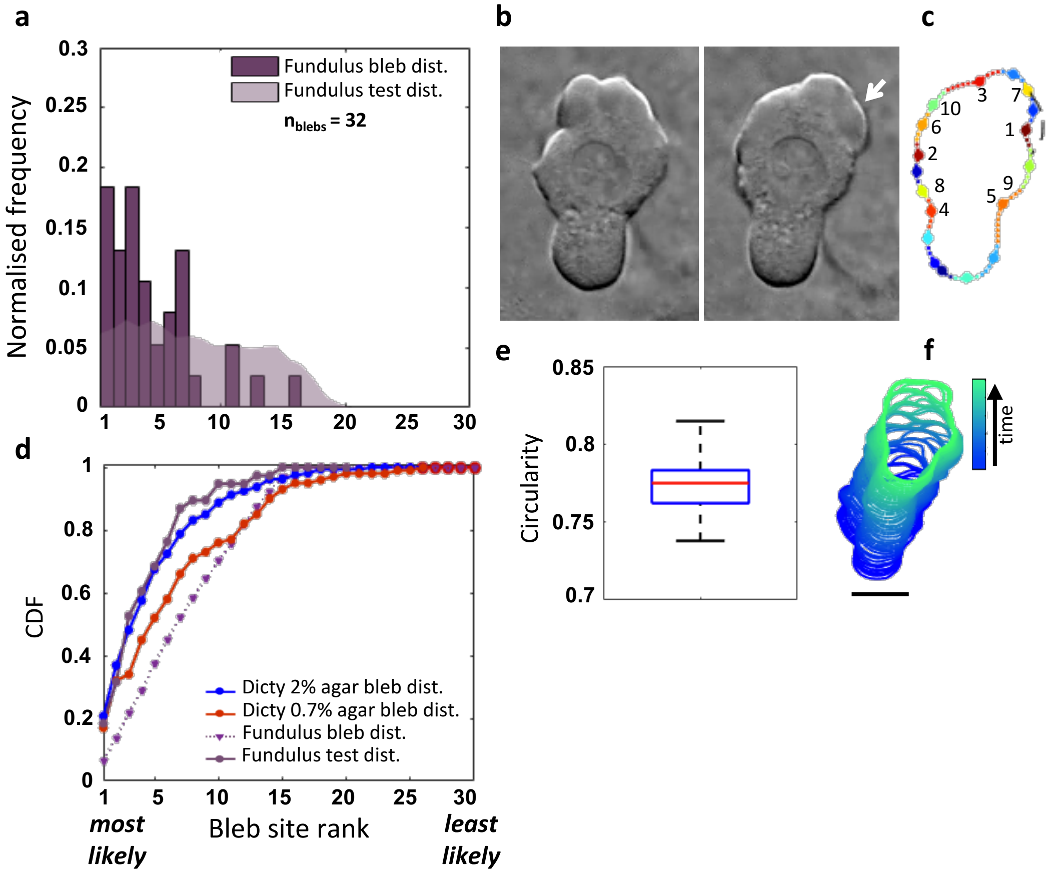

a
Normalised frequency
Fundulus bleb dist.
Fundulus test dist.
n_blebs = 32
b
c
d
CDF
Dicty 2% agar bleb dist.
Dicty 0.7% agar bleb dist.
Fundulus bleb dist.
Fundulus test dist.
most likely
Bleb site rank
least likely
e
Circularity
f
time

Figure SI 7
